# Jagged2 progressively increased expression from Stage I to III of Bladder Cancer and Melatonin-mediated downregulation of Notch/Jagged2 suppresses the Bladder Tumorigenesis *via* inhibiting PI3K/AKT/mTOR/MMPs signaling

**DOI:** 10.7150/ijbs.48358

**Published:** 2020-07-23

**Authors:** Yen-Ta Chen, Chi-Ruei Huang, Chia-Lo Chang, John Y. Chiang, Chi-Wen Luo, Hong-Hwa Chen, Hon-Kan Yip

**Affiliations:** 1 Division of Urology, Department of Surgery, Kaohsiung Chang Gung Memorial Hospital, Chang Gung University College of Medicine, Kaohsiung, Taiwan.; 2 Center for Shockwave Medicine and Tissue Engineering, Kaohsiung Chang Gung Memorial Hospital, Kaohsiung, Taiwan.; 3 Division of Cardiology, Department of Internal Medicine, Kaohsiung Chang Gung Memorial Hospital and Chang Gung University College of Medicine, Kaohsiung, Taiwan.; 4 Division of Colorectal Surgery, Department of Surgery, Kaohsiung Chang Gung Memorial Hospital and Chang Gung University College of Medicine, Kaohsiung, Taiwan.; 5 Department of Computer Science and Engineering, National Sun Yat-Sen University, Kaohsiung, Taiwan.; 6 Department of Healthcare Administration and Medical Informatics, Kaohsiung Medical University, Kaohsiung, Taiwan.; 7 Department of Surgery, Kaohsiung Medical University Hospital, Kaohsiung, Taiwan.; 8 Institute for Translational Research in Biomedicine, Kaohsiung Chang Gung Memorial Hospital Kaohsiung, Taiwan.; 9 Department of Medical Research, China Medical University Hospital, China Medical University, Taichung, Taiwan.; 10 Department of Nursing, Asia University Taichung, Taiwan.; 11 Division of Cardiology, Department of Internal Medicine, Xiamen Chang Gung Hospital, Xiamen, Fujian, China.

**Keywords:** Notch/Jagged2, bladder cancer, cell-stress signaling, matrix metalloproteinases

## Abstract

**Background:** This study assessed the expression of Jagged2 in human bladder cancer (BC) tested the hypothesis that melatonin (Mel) inhibited the tumorigenesis of BC cells mainly through downregulating the Notch/Jagged2 and PI3K/AKT/mTOR/MMPs^(2&9)^ signaling pathways.

**Methods and Results:** Tissue array from BC patients showed that the gene and protein expressions of JAG2/Jagged2 were significantly upregulated from T1 to T3 (primary tumor size) and from stage I to III (all p<0.001). *In vitro* study showed that in BC cell line of UMUC3, the cellular and protein expressions of Jagged2 were significantly attenuated in Mel-treated UMUC3 and further attenuated in UMUC3 shRNA silenced Notch/JAG2 (UMUC3^KD^) than in UMUC3 only (all p<0.0001). The protein expressions of Notch/Jagged2/MMPs^(2&9)^/PI3K/p-AKT/mTOR/p53/ratio of LC3BII/LC3B-I were significantly progressively reduced from UMUC3 to UMUC3+Mel/1.0mM, further to UMUC3+Mel/2.0mM and furthermore to UMUC3^KD^ (all p<0.0001). The cell proliferation/invasion/colony formation/healing-process were significantly inhibited in Mel-treated/2.0mM UMUC3 and further significantly inhibited in UMUC3^KD^ regardless of Mel treatment as compared with UMUC3 only (all p<0.0001). By day 28 after UMUC3 implanted into nude mouse back, the BC weight/volume were significantly reduced in UMUC3+Mel (100 mg/kg/day) and furthermore reduced in UMUC3^KD^ (all p<0.0001) as compared with UMUC3 only (all p<0.0001). The cellular (MMPs^(2&9)^/Notch/Jagged2) and protein (Notch/Jagged2/PI3K/p-AKT/mTOR/MMPs^(2&9)^) exhibited a similar trend, whereas the PTEN protein level exhibited an opposite pattern of PI3K among three groups (all p<0.0001).

**Conclusion:** Notch/Jagged-PI3K/p-AKT/mTOR/MMPs is one essential signaling pathway for BC survival, proliferation and invasion that were remarkably suppressed by Mel treatment.

## Introduction

Bladder cancer (BC) is the 9^th^ most common malignant tumor worldwide, with an estimate of more than 430,000 new cases and 165,000 deaths globally per year 1, 2. The patients are often lately diagnosed as an incurable invasive disease 3, 4. Transitional cell carcinoma (TCC), also known as urothelial carcinoma, is the most common type of bladder cancer which begins in urothelial cells that line the inside of the bladder.

Solid tumors are usually surrounded by distinctive structural barriers of basement membrane, a specialized thickening form of the extracellular matrix (ECM), that physically separate tumor from other tissue compartments 5. Metastasis represents a multi-step cell-biological process by which tumor cells disseminate from the primary tumor, invade through the basement membrane, spread into the circulatory system, and settle in secondary tissues where their growth continues 6. Metastasis is responsible of tumor recurrence in most cases, and more than 80% of TCC-related deaths are the result from secondary local or distant disease 7.

The Notch signaling pathway plays an important role in cell fate determination, proliferation, death, and cell-cell communication 8. Studies have further demonstrated that aberrant expression of Notch signaling was associated with a number of hematopoietic and epithelial human tumors including colorectal, prostate, skin, breast, cervical, lung cancers, leukemia, and neuroblastoma 9-16. Nevertheless, since the activation of Notch signaling upon the receptor-ligand engagement, the exact role of Notch activation in tumor microenvironment remains to be explored.

**Figure 1:**
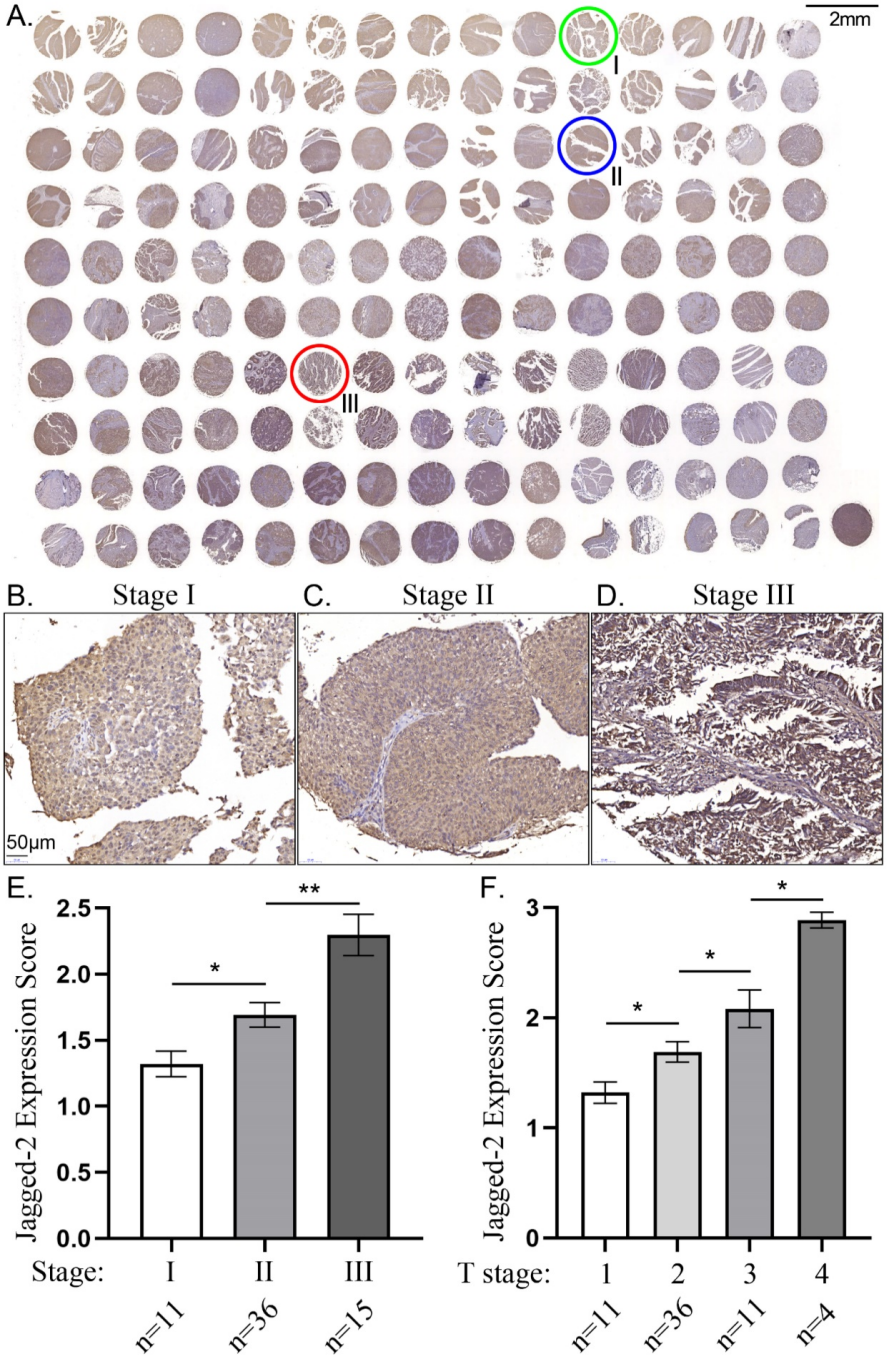
** The correlation between Jagged2 expression and tumor stage and primary tumor size in tissue array. A)** Illustrating immunohistochemical (IHC) staining of the tissue array for identification of Jagged2 expression. Green circle indicated the stage I bladder cancer (BC); Blue circle indicated stage II BC; Red circle indicated stage III BC. **B to D)** Showing the IHC stain for histopathologic finding for clarifying the BC in stage I (B), stage II (C) and stage III (D), respectively. **E)** Analytical result of intensity Jagged2 expression (i.e., scoring) in BC stages, * vs. stage I, P<0.01; ** vs. stage I, p<0.001. **F)** Analytical result of intensity Jagged2 expression (i.e., scoring) in primary tumor size, * vs. T1, P<0.01; ** vs. T1, p<0.001; *** vs. T1, p<0.0001.

**Figure 2:**
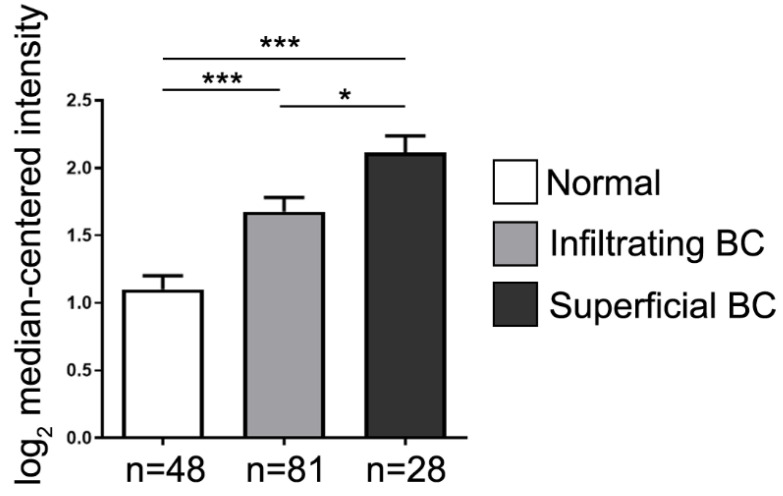
** The JAG2 gene expression in bladder cancer (BC).** Analytical result of JAG2 RNA expression was significantly higher in infiltrating type (i.e., localized in muscle layer) of BC and further higher in superficial type (i.e., invasion throughout the muscle layer) of BC than the normal control (NC). *** vs. NC, P<0.001; * vs. T1, p<0.05.

Study has previously demonstrated depletion of JAG2 gene intensely inhibited pancreatic cancer cell migration, invasion and metastasis without influencing cell proliferation 17. Presently, nearly 90% pancreatic cancer cells of Notch activation are identified to be almost always dependent upon Jagged2 ligand engagement 18. Activation of JAG2 (i.e., gene expression) in breast tumor cells induced EMT and cell survival 19. Jagged2 (i.e., protein expression) was also found to be significantly elevated in bone marrow stromal cells under hypoxia and promoted the self-renewal of cancer stem-like cells by activating Notch signaling 20. Additionally, the fact that expression status of Notch ligand Jagged2 was closely correlated with different grades of metastatic and recurrent bladder carcinoma suggested that Jagged2 plays an important role in tumor metastasis in bladder cancer 21. We have analyzed data from clinical research Oncomine database and tissue array demonstrating that JAG2 gen and Jagged2 protein expressions were upregulated in bladder cancer patients (refer to Figures 1 and 2). These evidences suggested that JAG2/Jgged2 could play an important role on bladder tumor proliferation and invasion.

Melatonin (Mel), synthesized in mitochondria and thus, most cells in human being can synthesize this small molecule 22, has been identified to be a robust antioxidant 23, 24 that strongly suppressed the formation of reactive oxygen species and attenuated inflammation. Plentiful data report that Mel protects against the development of malignancy 25-30 principally via its oncostatic features including potentiality to induce apoptosis, inhibit oxidative stress, draw forth immunomodulation as well as suppress angiogenesis and metastasis 31, 32. Furthermore, previous experimental studies have revealed that Mel could reduce migration and invasiveness of glioma, hepatoma, and breast cancer cells 33-35. These findings 23-35 suggest that Mel may play a critical role on regulating the BC cell migration and tumor growth.

## Materials and Methods

### Ethics statement

All animal procedures were approved by the Institute of Animal Care and Use Committee at Kaohsiung Chang Gung Memorial Hospital (Affidavit of Approval of Animal Use Protocol No. 2016122203) and performed in accordance with the Guide for the Care and Use of Laboratory Animals.

Animals were housed in an Association for Assessment and Accreditation of Laboratory Animal Care International (AAALAC; Frederick, MD, USA)-approved animal facility in our hospital with controlled temperature and light cycles (24 ^o^C and 12/12 light cycle).

### Animal grouping, time courses of Mel treatment and tumor size measurement

Pathogen-free, 12-week-old male nude mice (n = 30) weighing 22 - 25 g (Charles River Technology, BioLASCO, Taiwan) were equally categorized into three groups: group 1 [subcutaneous injection of UMUC3 cells, i.e., BC cell line (2.0 x 10^5^ cells) at the back of nude mouse], group 2 [subcutaneous injection of UMUC3 cells (2.0 x 10^5^ cells) at the back of nude mouse + Mel (100 mg/kg/day since days 8 to 24 after BC cell line injection for a total of 17 days) via intra-peritoneal injection] and group 3 [subcutaneous injection of UMUC3 cell line with knockdown (KD)^Notch/JAG2 gene^ (2.0 x 10^5^ cells) at the back of nude mouse].

The BC growth in living animals were measured (i.e., expressed as tumor volume with the value calculated as: width^2^ x length) at the time points of days 10, 14, 18, 21, 25 and 28 after the implantation of UMUC3 cell line in the left and right backs of nude mice.

The tumors were removed immediately after euthanizing the animals at day 28 after implantation of BC cell line of UMUC3. After tumor measurement, the specimens were cut into pieces and fixed with OCT (Tissue-Tek) for immunohistochemical (IHC) staining. The dosage of Mel was based on our previous report 36 with some modifications.

### shRNA clone transfection

shRNA clones were obtained from the National RNAi Core Facility Platform located at the Institute of Molecular Biology/Genomic Research Center, Academia Sinica, supported by the National Core Facility Program for Biotechnology Grants of NSC (NSC 100-2319-B-001-002). Individual clone was identified by its unique TRC number (e.g. sh*JAG2*: TRCN0000365342, sh*LacZ*: TRCN0000072233). Transient transfection of cells with plasmids was performed with Lipofectamine 3000 (Invitrogen, Life technologies, Carlsbad, CA, USA) according to the manufacturer's instructions with slight modifications. Briefly, 1 × 10^6^ cells were seeding to 10 cm plastic dish overnight. For use in transfection, 4 μl of Lipofectamine 3000 was incubated with 2 μg of indicated plasmid at room temperature. Cells were incubated at 37°C in a humidified atmosphere of 5% CO_2_. Following additional incubation of transfected cells overnight, transfected cells were selected with 1 μg/mL puromycin. qRT-PCR technique was performed to evaluate the efficiency of JAG2 silenced.

### siNotch1 transfection

Transient transfection of cells with siNotch1 was performed with Lipofectamine® *RNAiMAX* Transfection Reagent (Invitrogen, Life technologies, Carlsbad, CA, USA) according to the manufacturer's instructions with minimal modification. Briefly, 50% confluent cells were growing to 6-cm plastic dish overnight. For use in transfection, 18μl of Lipofectamine® *RNAiMAX* Transfection Reagent was incubated with 60 pmol of si*Notch1* at room temperature for 5 minutes. After the complex was added to the cells, the cells were incubated at 37°C in a humidified atmosphere of 5% CO_2_ for 72 hours. qRT-PCR technique was performed to evaluate the efficiency of Notch1 silenced.

### Cell culture

The BC cell lines of HT1197, HT1376, RT4 (Bioresource Collection and Research Center, Taiwan) and UM-UC-3 (i.e., UMUC3) [ATCC^®^ CRL-1749™ (American Type Culture Collection, USA)], and normal bladder cell line of SV-HUC-1 (Bioresource Collection and Research Center, Taiwan) were grown in Dulbecco's modified Eagle's medium (high glucose) supplemented with 10% fetal bovine serum and l% penicillin/streptomycin. All cultured cells were grown at 37°C and 5% CO_2_.

### MTT cell viability assay

Cell growth was determined by the MTT assay. About 2 × 10^3^ cells in 100 µL of medium were seeded into wells of a 96-well plate and incubated for the indicated duration. At the end of incubation, MTT solution was added into each well. After incubation, the purple crystal sediment was dissolved in DMSO and read at 540 nm in an ELISA reader. The absorbance value was used to represent the cell number.

### Assessment of colony formation

To determine the colony formation, 2000 cells per well were seeded in 6‐well dish with or without Mel (2.0 mM). After 7 day's seeding, the cells were fixed and permeabilized with methanol. Following washing twice with PBS, the colony was identified by microscope after 10% Giemsa solution (Sigma Corporation, Cream Ridge, NJ, USA) staining.

### Wound healing assay

For wound healing assay, 3.5 x 10^4^ cells were seeded in 12 wells with linear spacer inserts. Following overnight cell culture, a regular and defined “wound” within the cell monolayer was created by removing the linear spacer inserts. After phosphate‐buffered saline wash, the wells were either left untreated or treated with Mel (2.0 mM). After 24‐hour incubation, the migration of cells into the denuded areas in the marked region was monitored. The migration was captured with microphotography, and the total migration distance was determined using Image J software (National Institutes of Health, Bethesda, MD, USA).

### Transwell migration assay

Cells were first trypsinized and 5 × 10^4^ cells were then added to the Boyden chambers (8 µm pore size; Millipore, Billerica, MA, USA) with 0.5% FBS-containing medium and assay media that contained 10% FBS was added to the culture plates. After 24- hour incubation, the nonmotile cells at the top of the filter were removed and the motile cells at the bottom of the filter were fixed with methanol and stained with one‐tenth dilution of Giemsa (Sigma Corporation). The number of migrated cells in each chamber was carefully counted in five randomly chosen fields under the microscope for three independent experiments.

### Transwell invasion assay

Cells were plated in 0.5% FBS‐containing medium on the upper Boyden chamber coated with 100 μL of 10% Matrigel and 10% FBS‐containing medium in the lower chamber. Two days later, cells on the apical side of each insert were scraped off, and those of invading cells on the basolateral side of the membrane were fixed and stained the same as Transwell migration assay. The number of invading cells was counted in five randomly chosen fields under the microscope for three independent experiments.

### Western blot analysis

Equal amounts (30 µg) of protein extracts were separated by 8-12% SDS-PAGE. After electrophoresis, the separated proteins were transferred onto a polyvinylidene difiuoride (PVDF) membrane (Amersham Biosciences, Amersham, UK). Nonspecific sites were blocked by incubation of the membrane in blocking buffer [5% nonfat dry milk in T-TBS (TBS containing 0.05%Tween 20)] at room temperature for one hour. Then the membranes were incubated with the indicated primary antibodies [Notch (1:1000, Abcam), Jagged2 (1:1000, Signa Aldrich), matrix metalloproteinase (MMP)-2 (1:1000, Cell Signaling), MMP-9 (1:1000, Abcam), nuclear factor (NF)-κB (1:1000, Abcam), tumor necrosis factor (TNF)-α (1:1000, Cell Signaling), AKT (1: 1000, Cell Signaling), phosphorylated (p)-AKT (1: 1000, Cell Signaling), phospho-p38 (1: 1000, Cell Signaling), phospho-mTOR (1:1000, Cell Signaling), p53 (1:1000, Cell Signaling), cleaved caspase3 (1:1000, Cell Signaling), mitochondrial Bax (1:1000, Abcam), Bcl-2 (1:1000, biorbyt), Blc-xL (1:1000, Abcam), LC3B (1:1000, Cell Signaling), Cox IV (1:10000, Abcam) and GAPDH (1: 10000, Cell Signaling)] for 1 hour at room temperature. Horseradish peroxidase-conjugated anti-rabbit or anti-mouse immunoglobulin IgG (1:2000, Cell Signaling, Danvers, MA, USA) was used as a secondary antibody for one-hour incubation at room temperature. After being washed, the Immunoreactive membranes were visualized by enhanced chemiluminescence (ECL; Amersham Biosciences, Amersham, UK) and were exposed to medical X-ray film (FUAJI).

### Immunohistochemical (IHC) and immunofluorescent (IF) staining

In detail, for those of IHC and IF staining, rehydrated paraffin sections were first treated with 3% H_2_O_2_ for 30 minutes and incubated with Immuno-Block reagent (BioSB, Santa Barbara, CA, USA) for 30 minutes at room temperature. Sections were then incubated with primary antibodies specifically against MMP-2 (1:200, Invitrogen, CA, USA), MMP-9 (1:200, Invitrogen, CA, USA), Jagged2 (1:100, Abcam, Cambridge, UK) and Notch (1:150, Abcam, Cambridge, UK), while sections incubated with the use of irrelevant antibodies served as controls. Immunoreactive were visualized by enhanced DAB kit (Abcam).

### Statistical analysis

Quantitative data were expressed as mean ± SEM. Statistical analysis was adequately performed by ANOVA followed by Bonferroni multiple comparison post hoc test. SAS statistical software for Windows version 8.2 (SAS Institute, Cary, NC, USA) was utilized. A probability value <0.05 was considered statistically significant.

## Results

### The Jagged2 expression were significantly and progressively enhanced from T1 to T3 (i.e., primary tumor size) and from stage I to III

To elucidate whether a good correlation between the primary tumor size and the intensity of Jagged2 expression, the tissue array of human BC was utilized the study. As was expected, the Jagged2 expression the primary tumor was significantly progressively upregulated from T1 to T3 (**Figure 1**). Additionally, to further assess whether there also had a good association between the BC Stage and the intensity of Jagged2 expression, we further analysis with the tissue array. Consistently, the Jagged2 expression was significantly progressively augmented from stage I to III. These findings highlighted that Jagged2 protein could played a crucial role on BC growth, proliferation and invasion.

### The JAG2 gene expression in carcinoma of urogenital organ

To furthermore elucidate the JAG2 gene expression in urothelial carcinoma, data mining was performed from the cancer microarray database Oncomine 4.0 (Oncomine DB at http://www.oncomine.org) 37, 38. The result demonstrated that the JAG2 RNA expression (i.e., from microarray database) was significantly higher in infiltrating BC (i.e., TCC) and further higher in superficial BC than the normal control (**Figure 2**). This finding further supported that there had a strongly positive correlation between the Jagged2 expression and the BC stage.

### Protein and cellular expressions of Jagged2 in different kinds of bladder cancer cell lines and the protein expressions of cell-stress up- and down-stream signaling in UMUC3 cell line undergoing Mel treatment and Notch/JAG2 gene silence

**Figure 3:**
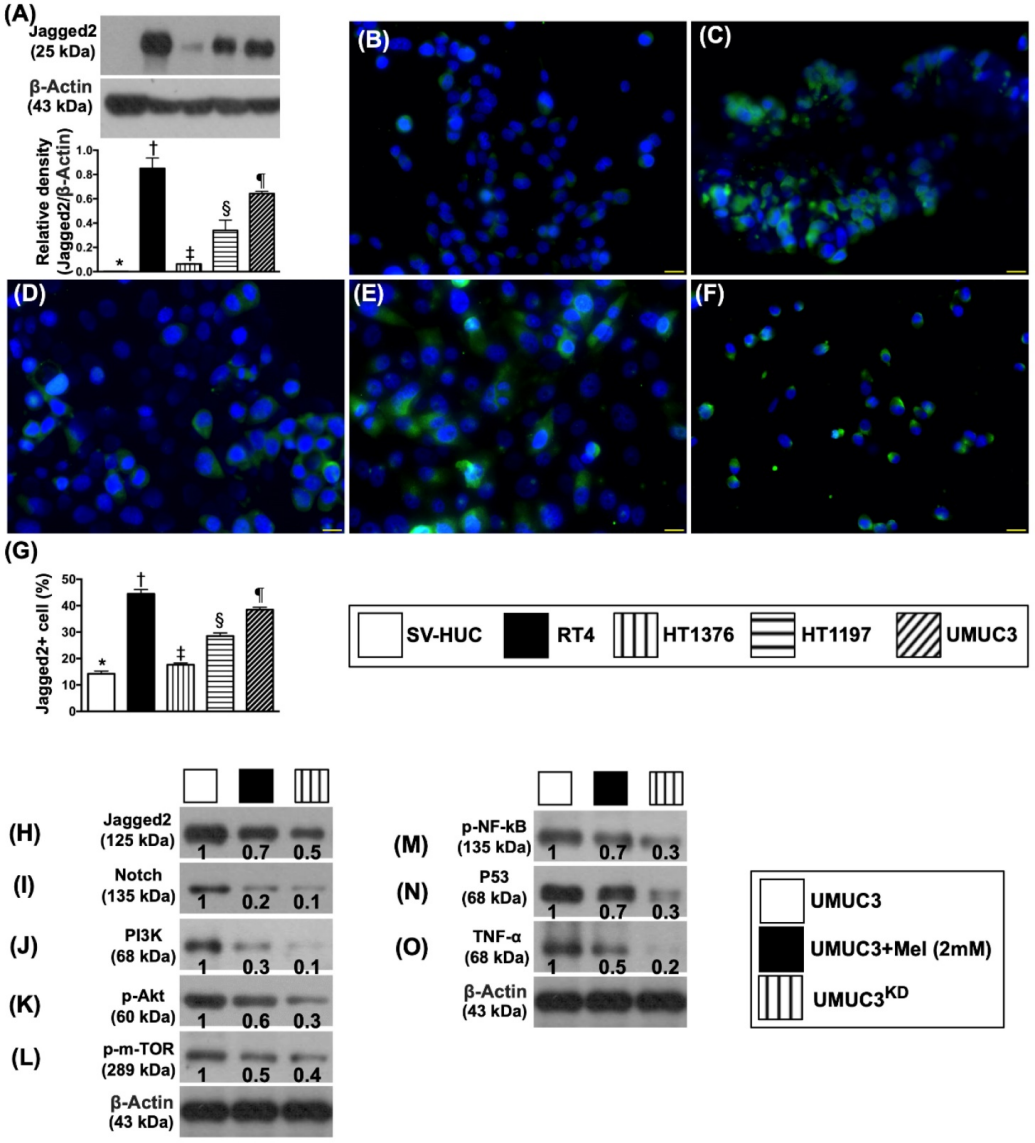
** Protein and cellular expressions of Jagged2 in different kinds of bladder cancer cell lines and the protein expressions of cell-stress up- and down-stream signaling in UMUC3 cell line undergoing Mel treatment and silenced Notch/JAG2 gene. A)** Protein expression of Jagged2, * vs. other groups with different symbols (†, ‡, §, ⁋), p<0.0001. **B to F)** Illustrating the immunofluorescent (IF) microscopic finding (400x) for identification of expression (%) of Jagged2+ cells (green color) in different bladder cancer cell lines. **G)** Analytical result of number of Jagged2+ cells, * vs. other groups with different symbols (†, ‡, §, ⁋), p<0.0001. All statistical analyses were performed by one-way ANOVA, followed by Bonferroni multiple comparison post hoc test (n=4 for each group). Symbols (*, †, ‡, §, ⁋) indicate significance (at 0.05 level). **H to O)** Protein expressions of Jagged2, Notch, PI3K, p-Akt, m-TOR, p-NF-κB, P53 and TNF-α were notably progressively reduced from UMUC3 to UMUC3 + Mel (2.0mM) and further to UMUC3^KD^, respectively. Actin indicates relative protein loading. Protein band density was quantified by densitometry, normalized to that of medium-treated control cells (which was set at 1.0), and was indicated below each Western blot. Mel = melatonin; SV-HUC1 = normal bladder cell line; UMUC3 = bladder cancer cell line; KD = knockdown; UMU3^KD^ = double KD of Notch/JAG2 gene (i.e., KD^Notch/JAG2^) in UMU3 cells.

According to the information of human BC (i.e., **Figures 1 and 2**), we designed *in vitro* and animal model study to further support the clinically relevant findings of tissue array and microarray database Oncomine. First, to elucidate the intrinsic protein expression of Jagged2 in normal bladder tissue and different BC cell lines (i.e., HT1376, HT1197, TR4, UMUC3), the protein expression of Jagged2 was examined. The result demonstrated that this parameter was lowest in SV-HUC (i.e., normal bladder tissue) and significantly progressively increased from HT1376 to HT1197 and furthermore increased in UMUC3 and RT4 (**Figure 3A**). Additionally, the cellular expression of Jagged2 showed an identical pattern of protein level among the groups (**Figure 3B-G**).

Second, to further investigate the key role of Notch/Jagged2 and therapeutic impact of Mel (2.0 mM) and gene knockdown (KD) of Notch/JAG2 (KD^Notch/JAG2^) (i.e., double KD) in UMUC3 cell line on the protein expressions of Jagged2/Notch and PI3K/p-AKT/mTOR, the Western blot analyses were performed. The preliminary results demonstrated that the protein expressions of PI3K/p-AKT/mTOR, an important signaling pathway for cell growth, differentiation, proliferation and survival, and Notch and Jagged2 were all prominently present in the UMUC3 cell line (**Figure 3H-L**). However, these parameters were remarkably reduced in Mel-treated UMUC3 cell line and furthermore remarkably reduced in KD^Notch/JAG2^ of UMUC3 cell line, suggesting that Notch/Jagged2 plays a principal role on BC growth, proliferation and invasion.

Consistently, the protein expression of p53 (**Figure 3M**), frequently activated in response to a stress signal for cell cycle arrest (i.e., a program that induces cell senescence or cellular apoptosis), displayed a similarly pattern of Jagged2 protein. Additionally, the protein expression of p-NF-κB (**Figure 3N**), crucial for control of transcription of DNA and cytokine production in responses to cell stress, also displayed a similar pattern of Jagged2. Finally, the protein expression of TNF-α, an indicator of pro-inflammatory cytokine, displayed an identical pattern of p-NF-κB (**Figure 3O**).

### Impact of stepwise increase in concentrations of Mel on protein expressions of Notch/Jagged2, matrix metalloproteinases (MMPs), PI3K/AKT/mTOR pathway, apoptosis and autophagy in UMUC3 cell line

**Figure 4:**
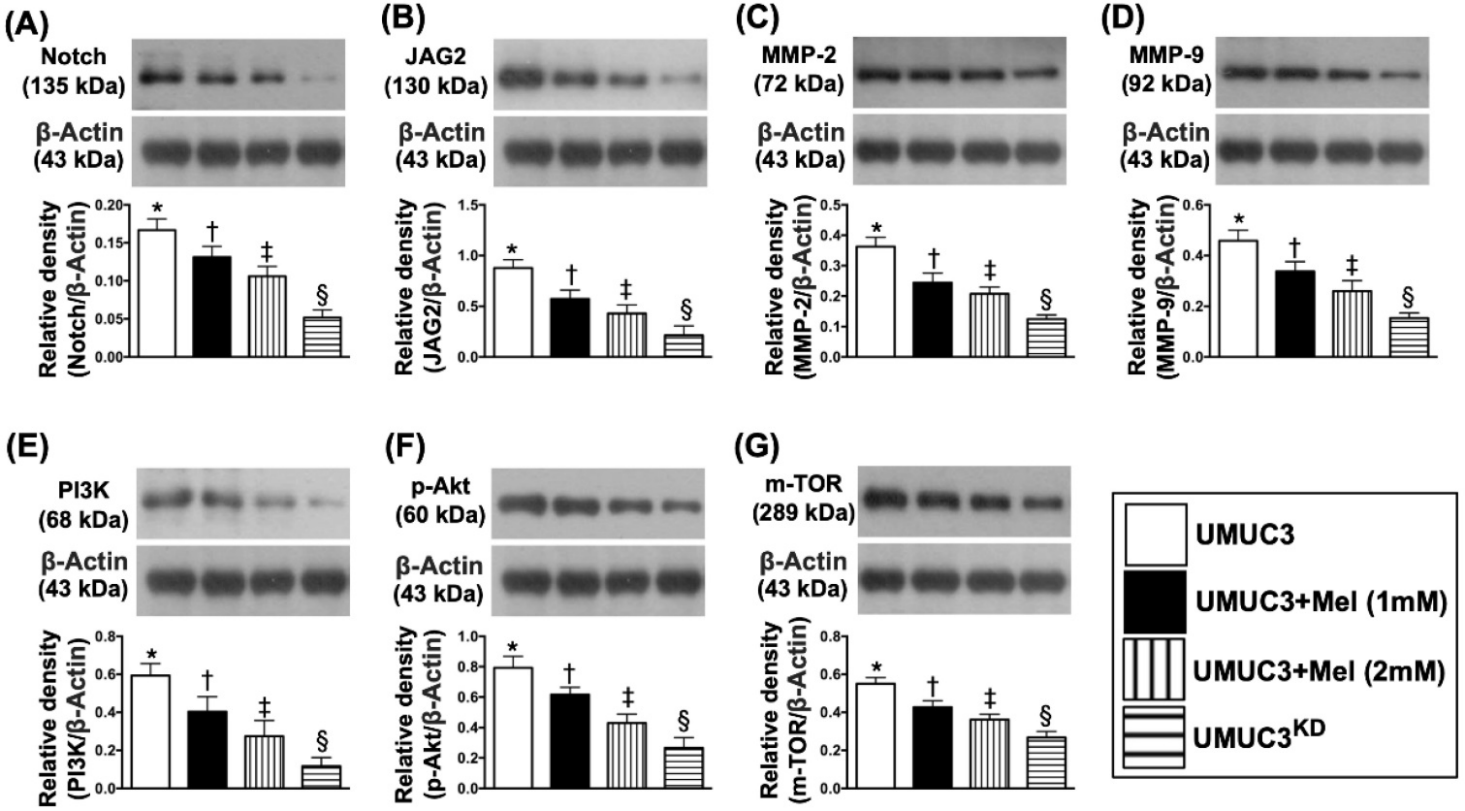
** Impact of stepwise increase in concentrations of Mel on protein expressions of Notch/Jagged2, matrix metalloproteinases (MMPs), PI3K/Akt/m-TOR pathway. A)** Protein expression of Notch, * vs. other groups with different symbols (†, ‡, §), p<0.0001. **B)** Protein expression of Jagged2, * vs. other groups with different symbols (†, ‡, §), p<0.0001. **C)** Protein expression of matrix metalloproteinase (MMP)-2, * vs. other groups with different symbols (†, ‡, §), p<0.0001. **D)** Protein expression of MMP-9, * vs. other groups with different symbols (†, ‡, §), p<0.0001. **E)** Protein expression of PI3K, * vs. other groups with different symbols (†, ‡, §), p<0.0001. **F)** Protein expression of phosphorylated (p)-Akt, * vs. other groups with different symbols (†, ‡, §), p<0.0001. **G)** Protein expression of mammalian target of rapamycin (m-TOR), * vs. other groups with different symbols (†, ‡, §), p<0.0001. All statistical analyses were performed by one-way ANOVA, followed by Bonferroni multiple comparison post hoc test (n=4 for each group). Symbols (*, †, ‡, §) indicate significance (at 0.05 level). Mel = melatonin; SV-HUC1 = normal bladder cell line; UMUC3 = bladder cancer cell line; KD = knockdown; UMU3^KD^ = double KD of Notch/JAG2 gene (i.e., KD^Notch/JAG2^) in UMU3 cells.

**Figure 5:**
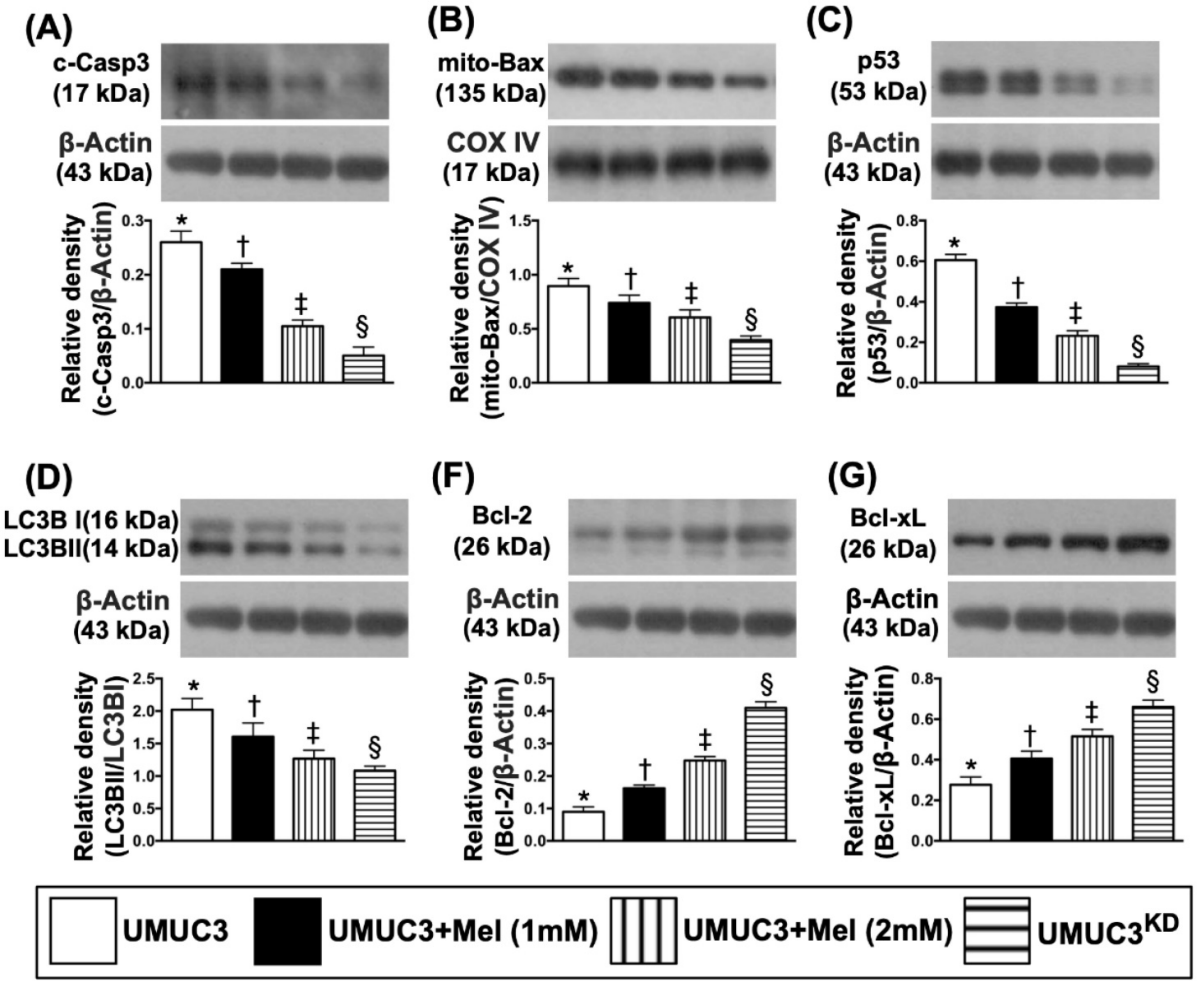
** Impact of stepwise increase in concentrations of Mel on protein expressions of apoptosis and autophagy in UMUC3 cells. A)** Protein expression of cleaved caspase 3 (c-Casp3), * vs. other groups with different symbols (†, ‡, §), p<0.0001. **B)** Protein expression of mitochondrial (mito)-Bax, * vs. other groups with different symbols (†, ‡, §), p<0.0001. **C)** Protein expression of p53, * vs. other groups with different symbols (†, ‡, §), p<0.0001. The ratio of LC3B-II/LC3B-I protein expression, * vs. other groups with different symbols (†, ‡, §), p<0.0001. **D)** Protein expression of Bcl-2, * vs. other groups with different symbols (†, ‡, §), p<0.0001. **E)** Protein expression of Blc-xL, * vs. other groups with different symbols (†, ‡, §), p<0.0001. All statistical analyses were performed by one-way ANOVA, followed by Bonferroni multiple comparison post hoc test (n=4 for each group). Symbols (*, †, ‡, §) indicate significance (at 0.05 level). Mel = melatonin; SV-HUC1 = normal bladder cell line; UMUC3 = bladder cancer cell line; KD = knockdown; UMU3^KD^ = double KD of Notch/JAG2 gene (i.e., KD^Notch/JAG2^) in UMU3 cells.

Based on the preliminary data in Figure 3, we further clarified the essential role of Mel on regulating the Notch/Jagged2 and its downstream signaling pathway by using the UMUC3 cell line culturing. The results showed that the protein expressions of Notch and Jagged2 were significantly and progressively reduced corresponding to the stepwise increased Mel concentration (i.e., 0, 1.0, 2.0 mM) and further significantly reduced in KD^Notch/JAG2^ (i.e., double KD) group (**Figure 4**). Additionally, the protein expressions of MMP-2 and MMP-9, crucial for tumor invasion, exhibited an identical pattern of Jagged2 among the four groups (Fig. 4). Furthermore, the protein expressions of PI3K/AKT/p-AKT/mTOR exhibited an identical pattern of Jagged2 among the four groups (**Figure 4**). Moreover, the protein expression of cleaved caspase 3, mitochondrial Bax and p53, three indicators of cell apoptosis/death, and ratio of LC3B-II/LC3B-1, an indicator of autophagic biomarker, showed an identical pattern of Jagged2 among the four groups (**Figure 5**). On the other hand, the protein expressions of Bcl-2 and Blc-xL, two indices of anti-apoptosis, exhibited an opposite pattern of Jagged2 among the four groups (**Figure 5**). These findings (**Figures 4 & 5**) implicated that PI3K/AKT/p-AKT/mTOR is the downstream signaling of Notch/Jagged2 and dependently regulated by Notch/Jagged2 signaling in UMUC3 cell line.

### Melatonin treatment and KD^Notch/JAG2^ gene suppressed proliferation/survival rate, wound healing process and colony formation of BC cells

To assess whether Mel treatment and KD^Notch/JAG2^ gene could suppress the proliferation rate, wound healing and colony formation of BC cells, the UMUC3 cell line was utilized in the *in vitro* study.

**Figure 6:**
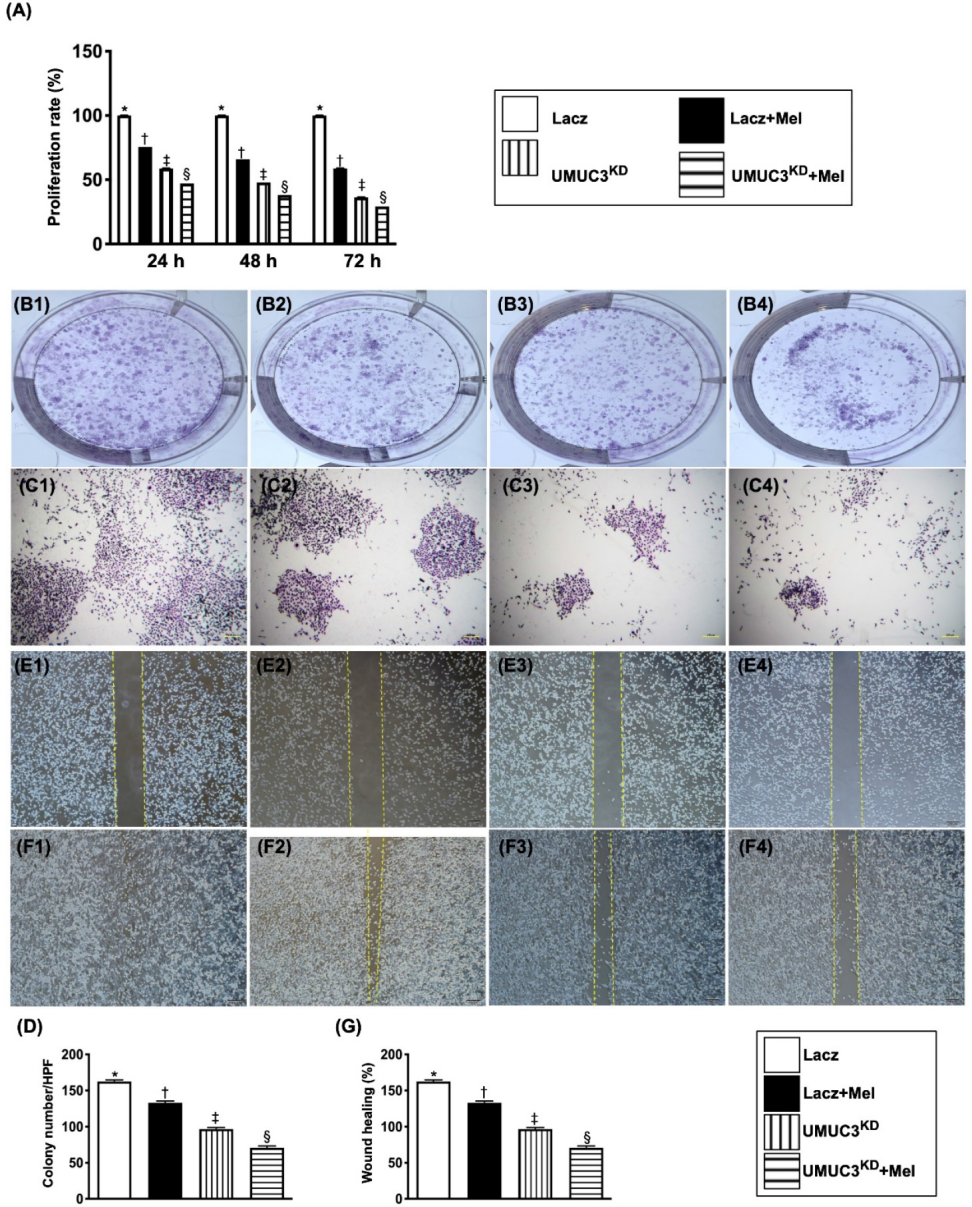
** Melatonin treatment and double KD^Notch/JAG2^ gene suppressed proliferation/survival rate, colony formation and migration of UMUC3 cells. A)** Illustrating the results of MTT assay for determining the proliferation/survival rate of UMUC3 cells under Mel treatment (2.0 mM) or in condition of KD^Notch/JAG2^ gene expression, by 24, 48 and 72h after cell culture, respectively. * vs. other groups with different symbols (†, ‡, §), p<0.001 at 24h (green bar chart), at 48h (yellow bar chart) and 72 (blue bar chart) cell culturing respectively. **B1 to B4 and C1 to C4)** Illustrating the microscopic finding at 0h (i.e., B1 to B4) (1x, in culture plate) and 24h (i.e., C1 to C4) (40x) for identification of the colony formation (cell numbers: 2 x 10^3^/well) in the four groups. **D)** Analytical result, * vs. other groups with different symbols (†, ‡, §), p<0.001. **E1 to E4 and F1 to F4)** Illustrating the microscopic finding (40x) at 0h (i.e., E1 to E4) and 24h (i.e., F1 to F4) for identification of “wound healing” process (cell amount: 3.5 x 10^4^/well) among the four groups. **G)** Analytical results % of wound healing at 24h, * vs. other groups with different symbols (†, ‡, §), p<0.001. The formula for calculation of wound healing process (%) = cell migrated area at 24h/the original migrated area at 0h. All statistical analyses were performed by one-way ANOVA, followed by Bonferroni multiple comparison post hoc test (n=3 for each group). Symbols (*, †, ‡, §) indicate significance (at 0.05 level). Lacz = control; Mel = melatonin; UMUC3 = bladder cancer cell line; KD = knockdown; UMU3^KD^ = double KD of Notch/JAG2 gene (i.e., KD^Notch/JAG2^) in UMU3 cells.

As we expected, the flow cytometric analysis showed that as compared with that of the control group (i.e., medium-treated UMUC3), the proliferation rate of these BC cells was significantly inhibited by Mel (2.0 mM) and furthermore significantly inhibited in KD^Notch/JAG2^ plus Mel treatment in time intervals from 0, 12, 48 to 72 h (**Figure 6**).

Consistently, the colony formation and healing process of UMUC3 cells were significantly inhibited by Mel and further significantly inhibited by KD^Notch/JAG2^ gene regardless of Mel treatment as compared to those of the control group.

### Mel treatment (2.0 mM) and KD^Notch/JAG2^ gene in UMUC3 cell line inhibited invasion ability and attachment to endothelial cells

**Figure 7:**
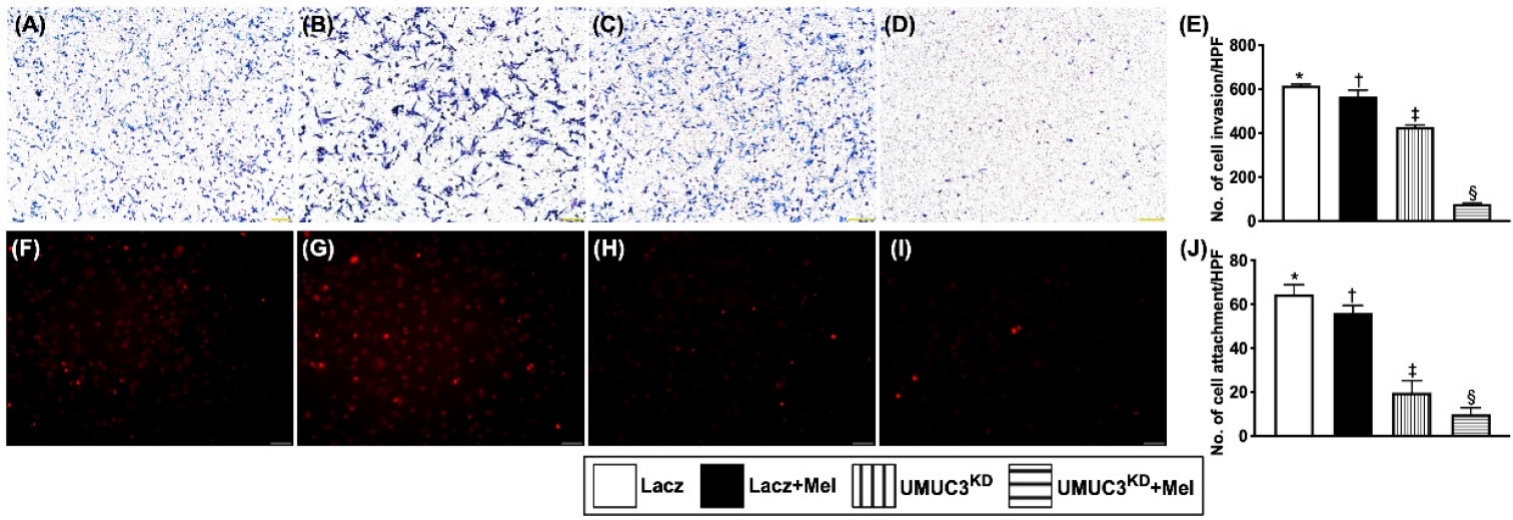
** Mel treatment (2.0 mM) and Silenced Notch/JAG2 gene in UMUC3 cell line inhibited invasion ability and attachment to endothelial cells. A-D)** Illustrating the microscopic finding (40x) of Giemsa staining for identification of cell invasion among four groups. **E)** Analytical result of invasion ability of UMUC3 cells (cell amount 2 x 10^3^/well), * vs. other groups with different symbols (†, ‡, §), p<0.001. **F-I)** Illustrating the microscopic finding (200x) for identification of the attachment capacity of UMUC3 cells (cell amount 1 x 10^5^/well) to human umbilical vein-derived endothelial cells (HUVECs) (cell amount 8 x 10^4^/well). Red color indicates the Dil dye stained the UMUC3 cells**. J)** Number of UMUC3 cell attachment, * vs. other groups with different symbols (†, ‡, §), p<0.001. All statistical analyses were performed by one-way ANOVA, followed by Bonferroni multiple comparison post hoc test (n=3 for each group). Symbols (*, †, ‡, §) indicate significance (at 0.05 level). Lacz = control; Mel = melatonin; UMUC3 = bladder cancer cell line; KD = knockdown; UMU3^KD^ = double KD of Notch/JAG2 gene (i.e., KD^Notch/JAG2^) in UMU3 cells.

The invasive capacity of BC cells was examined using an invasion assay (i.e., UMUC3 cells were seeded in Matrigel-containing Transwell). Following 72 hours of incubation, cell invasion was examined microscopically after Giemsa staining. The results showed that, as compared with that in the control group, the invasion ability of UMUC3 cells was significantly reduced by Mel treatment (2.0 mM) and further significantly inhibited by KD^Notch/JAG2^ gene regardless of Mel treatment (**Figure 7**). Besides, the capacity of attachment of UMUC3 cells to endothelial cells was also significantly suppressed compared to that in the control group regardless of Mel treatment. Our findings from **Figures 6 and 7** highlighted the Notch/Jagged2 pathway is crucial for proliferation, growth and invasion of BC cells.

### The Mel treatment suppressed the protein expressions of matrix metalloproteinases and cell-stress signaling pathway in bladder tumor of UMUC3 cell line formation in nude mice

**Figure 8:**
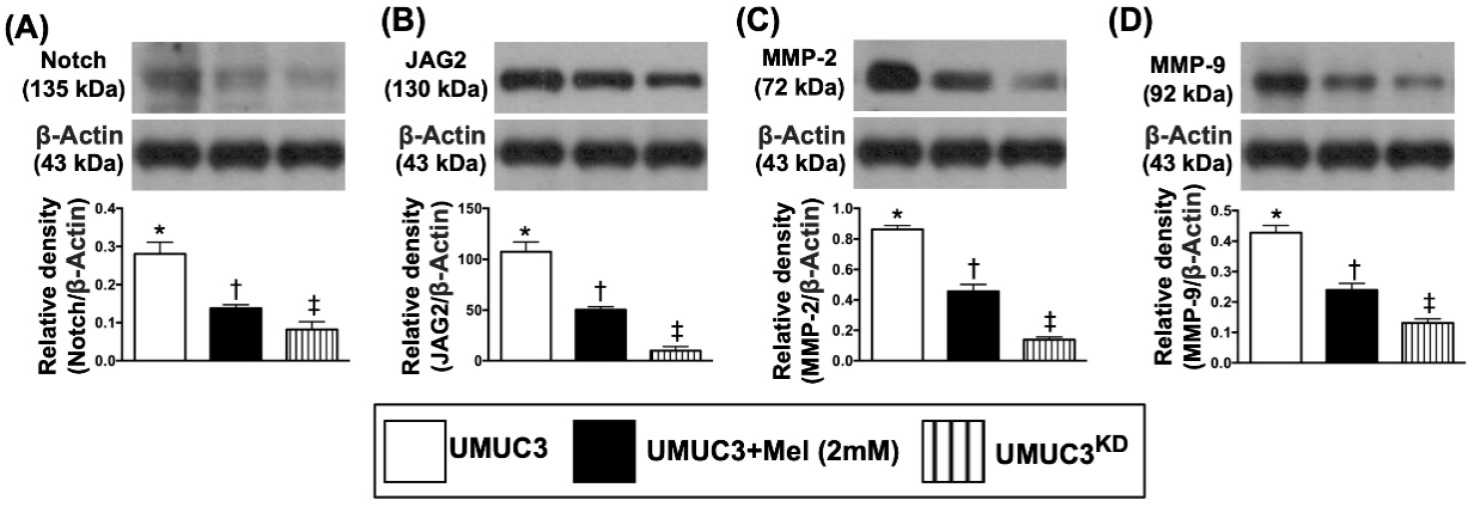
** Mel treatment suppressed the protein expressions of Notch, Jagged2 and MMPs in bladder tumor harvested from nude mouse back. A)** Protein expression of Notch, * vs. other groups with different symbols (†, ‡), p<0.0001. **B)** Protein expression of Jagged2, * vs. other groups with different symbols (†, ‡), p<0.0001. **C)** Protein expression of matrix metalloproteinase (MMP)-2, * vs. other groups with different symbols (†, ‡), p<0.0001. **D)** Protein expression of MMP-9, * vs. other groups with different symbols (†, ‡), p<0.0001. All statistical analyses were performed by one-way ANOVA, followed by Bonferroni multiple comparison post hoc test (n=6 for each group). Symbols (*, †, ‡, §) indicate significance (at 0.05 level). Mel = melatonin; UMUC3 = bladder cancer cell line; KD = knockdown; UMU3^KD^ = double KD of Notch/JAG2 gene (i.e., KD^Notch/JAG2^) in UMU3 cells.

By day 28, the bladder tumor (UMUC3 cell line) mass was harvested from nude mouse back for Western blot examination. The results demonstrated that the protein expressions of Notch and Notch ligand Jagged2, the key signaling pathway of BC growth, proliferation and metastasis, were significantly reduced in BC + Mel (i.e., group 2) than in BC (i.e., group 1), and were even more significantly reduced in BC + KD^Notch/JAG2^ (i.e., group 3) (**Figure 8**). Additionally, the protein expressions of MMP-2 and MMP-9, two proteolytic enzymes of matrix metalloproteinases (MMPs) frequently playing a critical role for tumor invasion and distal metastasis, exhibited an identical pattern of Jagged2 among the three groups (**Figure 8**).

**Figure 9:**
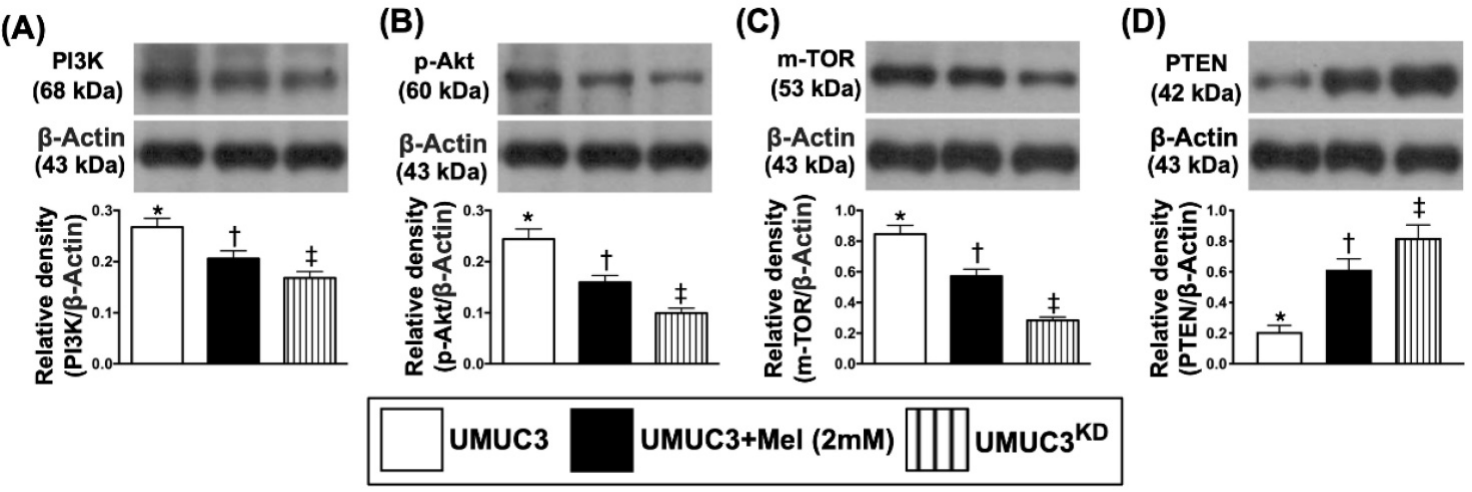
** Mel treatment suppressed the protein expressions of cell-stress signaling and apoptosis in bladder tumor harvested from nude mouse back. A)** Protein expression of PI3K, * vs. other groups with different symbols (†, ‡), p<0.0001. **B)** Protein expression of phosphorylated (p)-AKT, * vs. other groups with different symbols (†, ‡), p<0.0001. **C)** Protein expression of mTOR, * vs. other groups with different symbols (†, ‡), p<0.0001. **D)** Protein expression of PTEN, * vs. other groups with different symbols (†, ‡), p<0.0001. All statistical analyses were performed by one-way ANOVA, followed by Bonferroni multiple comparison post hoc test (n=6 for each group). Symbols (*, †, ‡, §) indicate significance (at 0.05 level). Mel = melatonin; KD = knockdown; UMUC3 = bladder cancer cell line; UMU3^KD^ = double KD of Notch/JAG2 gene (i.e., KD^Notch/JAG2^) in UMU3 cells.

The protein expressions of PI3K (i.e., a positive BC regulator), p-AKT and mTOR, the signaling pathway of cell survival, growth and proliferation, demonstrated an identical pattern of Jagged2 among the three groups (**Figure 9**). On the other hand, the protein expression of PTEN, a negative BC regulator, showed an opposite pattern of PI3K among the three groups (**Figure 9**).

### Time courses of tumor volume and tumor weight by day 28 after UMUC3 cell implantation in nude mice

**Figure 10:**
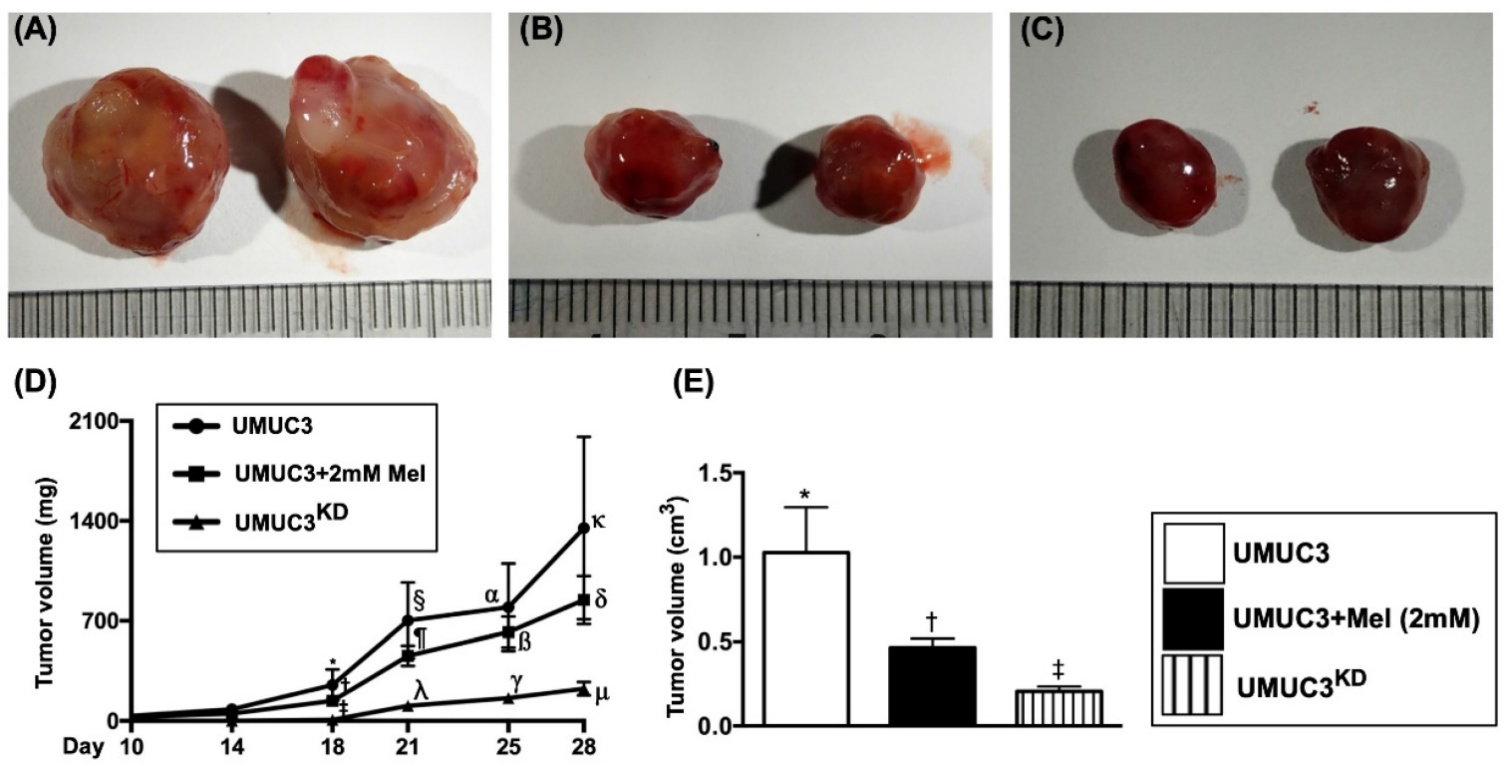
** Time courses of tumor volume and tumor weight by day 28 after UMUC3 cell implantation in nude mice. A-C)** Demonstrating the grossly anatomical feature of tumor mass in UMUC3 (A), UMUC3 +Mel (2.0 mM) (B) and UMU3^KD^ (C) groups, respectively. The tumor size was notably reduced in UMUC3 +Mel and further notably reduced in UMU3^KD^ as compared with UMUC3 only. **D)** Graphically illustrated the analytic results of the time courses of growth volume of bladder tumor at days 14, 18, 21, 25 and 28. By day 18, * vs. other groups with different symbols (†, ‡), p<0.01. By day 21, § vs. other groups with different symbols (⁋, λ), p<0.001. By day 25, α vs. other groups with different symbols (ß, γ), p<0.0001. By day 28, κ vs. other groups with different symbols (δ, μ), p<0.0001. **E)** By day 28, the mean harvested bladder cancer weight, * vs. other groups with different symbols (†, ‡), p<0.0001. All statistical analyses were performed by one-way ANOVA, followed by Bonferroni multiple comparison post hoc test (n=8 for each group). Symbols (*, †, ‡, §) indicate significance (at 0.05 level). Mel = melatonin; KD = knockdown; UMUC3 = bladder cancer cell line; UMU3^KD^ = double KD of Notch/JAG2 gene (i.e., KD^Notch/JAG2^) in UMU3 cells.

To assess the tumor growth in the living animals, the time points of measurement of tumor size were at days 10, 14, 18, 21, 25 and 28 after the implantation of UMUC3 cell line in the left and right backs of nude mice. The results demonstrated that the tumor volume was significantly progressively increased in group 1 from day 10 to day 28 (**Figure 10**). However, as compared to the group 1, the tumor volume was significantly reduced in group 2 and further significantly reduced in group 3 in these time intervals. Additionally, by day 28 (i.e., the end of study period) the harvested tumor weight was also significantly reduced in group 2 and further significantly reduced in group 3 than in group 1.

### The cellular expressions of matrix metalloproteinases and Notch/Jagged2 in harvested tumor by day 28 after UMUC3 cell implantation

**Figure 11:**
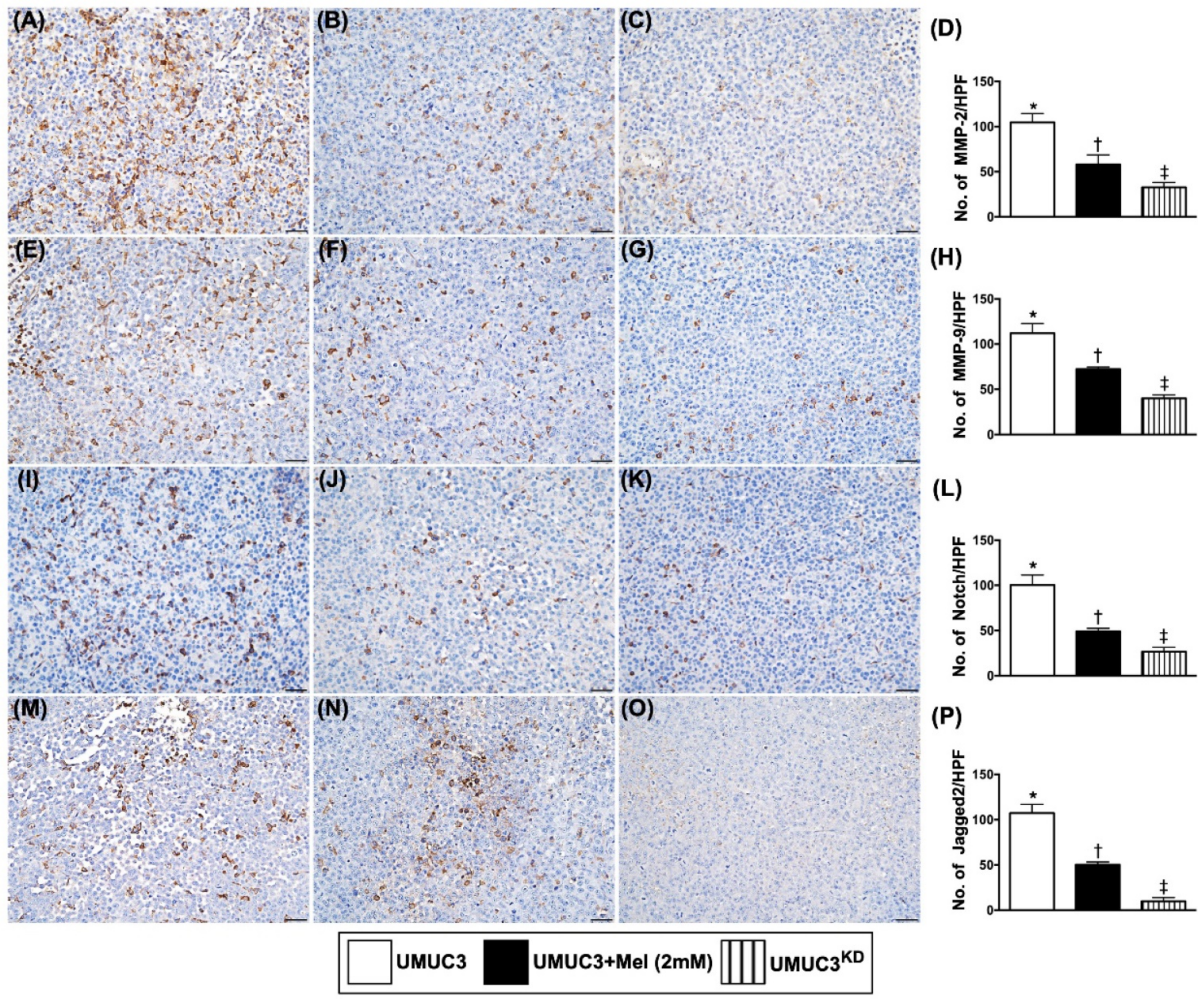
** Cellular expressions of matrix metalloproteinases and Notch/Jagged2 in harvested bladder cancer tissue by day 28 after UMUC3 cell implantation into nude mouse back. A-C)** Illustrating the microscopic finding (200x) for identification of cellular infiltration of matrix metalloproteinase (MMP)-2 in bladder cancer tissue (gray color). **D)** Analytical result of number of MMP-2+ cells, * vs. other groups with different symbols (†, ‡), p<0.0001. **E-G)** Illustrating the microscopic finding (200x) for identification of cellular infiltration of MMP-9 in bladder cancer tissue (gray color). **H)** Analytical result of number of MMP-9+ cell, * vs. other groups with different symbols (†, ‡), p<0.0001. **I-K)** Illustrating the microscopic finding (200x) for identification of cellular expression of Notch in bladder cancer tissue (gray colour). **L)** Analytical result of number of Notch+ cells, * vs. other groups with different symbols (†, ‡), p<0.0001. **M-O)** Illustrating the microscopic finding (200x) for identification of cellular expression of Jagged2 in bladder cancer tissue (gray color). **P)** Analytical result of number of Jagged2+ cells, * vs. other groups with different symbols (†, ‡), p<0.0001. All statistical analyses were performed by one-way ANOVA, followed by Bonferroni multiple comparison post hoc test (n=6 for each group). Symbols (*, †, ‡, §) indicate significance (at 0.05 level). Mel = melatonin; UMUC3 = bladder cancer cell line; UMU3^KD^ = double KD of Notch/JAG2 gene (i.e., KD^Notch/JAG2^) in UMU3 cells.

The cellular infiltrations of MMP-2 and MMP-9, two indicators of matrix metalloproteinases, were significantly suppressed in group 2 and further significantly suppressed in group 3 than in those of the group 1 (**Figure 11**). Additionally, the cellular expressions of Notch and Jagged2, displayed an identical pattern to MMPs among the three groups.

## Discussion

**Figure 12:**
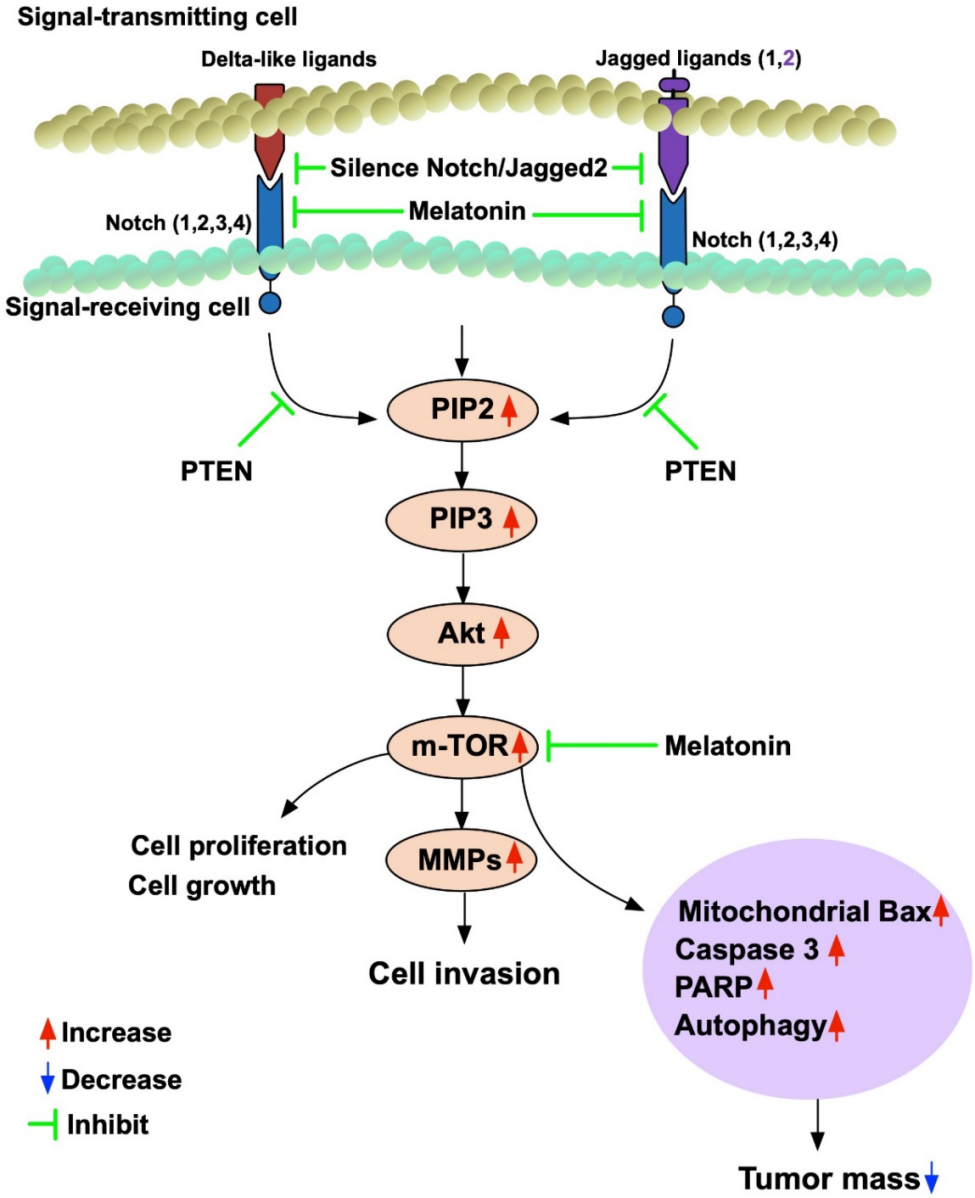
Schematically drawing the diagraph for illustrating the signaling pathway and pointing the action sites of melatonin. MMPs = matrix metalloproteinases.

This study which investigated the impact of Mel and Notch/Jagged2 signaling pathway on regulating the BC growth, proliferation, differentiation and invasion yielded several striking implications. First, the histopathological findings (i.e., IHC staining) from tissue array and gene expression for Oncomine database 37, 38 of BC patients demonstrated that there is a strong positive correlation between the intensity of Jaaged2/JAG2 expression and the stage I to III as well as T1 to T3 of BC. Second, *in vitro* study demonstrated that Notch/Jagged2 signaling played a crucial role on regulating the proliferation/differentiation, growth and invasion of BC. *In vitro* and *in vivo* consistently demonstrated that Mel treatment significantly suppressed the proliferation/differentiation, growth and invasion of BC through downregulating the signaling pathway (i.e., PI3K/AKT/mTOR). Additionally, silenced Notch/JAG2 gene of BC cells, regardless of Mel treatment, was superior to Mel treatment for inhibiting the PI3K/AKT/mTOR signaling pathway, which in turn, suppressed the bladder tumor growth. Finally, based on the results of our study, we have schematically drawn a diagraph (**Figure 12**) to illustrate the signaling pathway and point the action sites of Mel for more easily reading the findings from the current study.

Previous studies have established that interaction between Notch and Jagged2 was crucial in cancer cell migration, invasion, metastasis and proliferation 17-19. Additionally, Notch ligand Jagged2 was tightly linked with different grades of metastatic and recurrent BC 21. One important finding from tissue array and Oncomine database demonstrated that the Jagged2/JAG2 expressions were notably highly associated with an increase in BC stage and primary tumor size as well as BC invasion, highlighting that the findings from previous studies 17-19, 21 and tissue array/Oncomine database were consistent with each other.

Based on these above-mentioned issues, we further performed an *in vitro* study (referred to in **Figures 3-5**). Importantly, the result revealed that not only Mel treatment significantly suppressed, but also silenced Notch/JAG2 gene further significantly suppressed the protein expressions and the PI3K/AKT/mTOR signaling in UMUC3 cells inevitably leading us to raise the hypothesis that upstream signaling of Notch/JAG2 might play a principal role on the growth, proliferation and invasion/metastasis of BC essentially through upregulating the PI3K/AKT/mTOR downstream signaling.

Interestingly, our previous study demonstrated that Mel treatment mediated downregulation of ZNF746 suppresses bladder tumorigenesis mainly through inhibiting the AKT-MMP-9 signaling pathway 39. Based on our hypothesis and using the cell culturing, we further identified that Mel treatment markedly inhibited the colony formation, cell survival rate and invasion ability of UMUC3 cells as well as the ability of these cancer cells attached to endothelial cells (referred to in **Figures 6 and 7**). The principal finding of these *in vitro* studies was that silenced Notch/JAG2 gene of these cancer cells furthermore markedly inhibited the colony formation, cell survival rate and invasion ability of UMUC3 dependently through mediating the cell survival signaling pathway of PI3K/AKT/mTOR signaling regardless of the Mel treatment. Our finding is compatible with the findings of our previous report 39.

Finally, to further confirm the axis of Notch/Jaggged2-MMPs-PI3K/AKT/mTOR signaling played a unique role on participating the BC, an animal model of study was utilized. As our expectation, the protein expressions of Jagged2, Notch, MMP-2/MMP-9 and PI3K/AKT/mTOR signaling were significantly reduced by Mel treatment and further significantly reduced by silenced *Notch/JAG2* in the harvested tumor mass. Additionally, the cellular levels of MMP-2/MMP-9 and Notch/Jagged2 also expressed similarity to the protein level of Jagged2. These findings could explain why the tumor volume and tumor weight were substantially reduced in Mel-treated group and further substantially depressed by silenced Notch/*JAG2* gene group. Our recent study 39 has demonstrated that the intracellular and mitochondrial reactive oxygen species was remarkably increased in cancer cells as compared with those of normal cells. Intriguingly, another recent study has elucidated novel information that Mel owns its anticancer effects that might be mediated by regulating the mitochondria 22. In this way, the findings from these two studies 22, 39 strengthen the results of our study.

Finally, it is worthy to discuss the finding of protein expression of p53 which was notably suppressed by Mel treatment and furthermore suppressed by silenced* Notch/JAG2* in UMUC3 cells. Basic research has previously clearly demonstrated that p53 acts as a tumor suppressor 40. Additionally, the p53 pathway which always constitutes positive and negative feedback loops responds to stresses that can disrupt the allegiance of DNA replication and cell division 41. Thus, p53 plays a key role on tumor cell apoptosis and death 41. We remain uncertain for why the protein expression of p53 was comparable with the alternative pattern of apoptosis (i.e., mitochondrial Bax, caspase 3). Perhaps, it could be due to the activity of the UMUC3 cells was remarkably suppressed by Mel treatment or by silenced* Notch/JAG2,* i.e., just a negative feedback phenomenon 41.
